# Innate Immune Responses to Bacterial Ligands in the Peripheral Human Lung – Role of Alveolar Epithelial TLR Expression and Signalling

**DOI:** 10.1371/journal.pone.0021827

**Published:** 2011-07-15

**Authors:** Andrew J. Thorley, Davide Grandolfo, Eric Lim, Peter Goldstraw, Alan Young, Teresa D. Tetley

**Affiliations:** 1 Lung Cell Biology, Section of Pharmacology and Toxicology, National Heart and Lung Institute, Imperial College, London, United Kingdom; 2 Department of Thoracic Surgery, Royal Brompton and Harefield NHS Trust, London, United Kingdom; 3 AstraZeneca R&D, Loughborough, United Kingdom; University of Pittsburgh, United States of America

## Abstract

It is widely believed that the alveolar epithelium is unresponsive to LPS, in the absence of serum, due to low expression of TLR4 and CD14. Furthermore, the responsiveness of the epithelium to TLR-2 ligands is also poorly understood. We hypothesised that human alveolar type I (ATI) and type II (ATII) epithelial cells were responsive to TLR2 and TLR4 ligands (MALP-2 and LPS respectively), expressed the necessary TLRs and co-receptors (CD14 and MD2) and released distinct profiles of cytokines via differential activation of MAP kinases. Primary ATII cells and alveolar macrophages and an immortalised ATI cell line (TT1) elicited CD14 and MD2-dependent responses to LPS which did not require the addition of exogenous soluble CD14. TT1 and primary ATII cells expressed CD14 whereas A549 cells did not, as confirmed by flow cytometry. Following LPS and MALP-2 exposure, macrophages and ATII cells released significant amounts of TNFα, IL-8 and MCP-1 whereas TT1 cells only released IL-8 and MCP-1. P38, ERK and JNK were involved in MALP-2 and LPS-induced cytokine release from all three cell types. However, ERK and JNK were significantly more important than p38 in cytokine release from macrophages whereas all three were similarly involved in LPS-induced mediator release from TT1 cells. In ATII cells, JNK was significantly more important than p38 and ERK in LPS-induced MCP-1 release. MALP-2 and LPS exposure stimulated TLR4 protein expression in all three cell types; significantly more so in ATII cells than macrophages and TT1 cells. In conclusion, this is the first study describing the expression of CD14 on, and TLR2 and 4 signalling in, primary human ATII cells and ATI cells; suggesting that differential activation of MAP kinases, cytokine secretion and TLR4 expression by the alveolar epithelium and macrophages is important in orchestrating a co-ordinated response to inhaled pathogens.

## Introduction

The respiratory tract is one of the primary routes of entry to the body for invading pathogens. As such, it requires tightly regulated mechanisms with which to respond to a potentially endless spectrum of microbes. One line of defence is the expression of a family of receptors known as the Toll-like receptors (TLRs) that are able to recognise a variety of microbial markers. At present 11 receptors have been identified in man, each of which recognises distinct pathogen-associated molecular patterns (PAMPs) [1]. These receptors can be divided by the source of the PAMPs that they recognise. Thus, TLRs 1, 2, 4, 5 and 6 recognise largely bacterial pathogens, whereas TLRs 3, 7 and 8 recognise predominantly viral motifs [2].

TLR-2 recognises a broad spectrum of microbial products such as peptidoglycan from gram-positive bacteria, bacterial lipoproteins, lipoarabinomannan from mycobacteria and yeast cell walls. Its broad range of ligand specificity may be accountable to its ability to heterodimerise with two other TLRs, TLR-1 [3] and TLR-6 [4]. In this way, these heterodimers confer specificity for a certain subset of the aforementioned ligands. For example, the TLR-2/6 heterodimer specifically recognises the mycoplasmal lipoprotein, macrophage-activating lipopeptide 2 kDa (MALP-2). This selectivity has been shown to be due to the ability of TLR-6 to differentiate between triacylated bacterial lipoproteins and the diacylated MALP-2. In mice lacking TLR-2, macrophages were unresponsive to both bacterial and mycoplasmal lipoproteins whereas TLR-6-deficient mice were only unresponsive to MALP-2 [4].

TLR4 is the primary receptor for recognition of lipopolysaccharide (LPS), found on gram negative bacteria, and was the first mammalian TLR to be characterised [5]. TLR4 signalling requires a number of accessory proteins to initiate a signal. A key component of the TLR4 receptor cluster is MD-2. MD-2 is a soluble protein that binds to the extracellular domain of TLR4 and is essential for LPS recognition [6]. Studies of the receptor structure have demonstrated that MD-2 binds directly to LPS causing an allosteric change in that facilitates its binding to a second TLR4 protein thus forming a receptor dimer [7], [8]. These studies also suggest that LPS does not directly bind to the TLR4 receptor, thus explaining the obligate requirement for MD-2.

Another important TLR4 accessory protein is CD14, a high affinity receptor that is found on the surface of cells such as macrophages and monocytes [9] in a GPI-anchored form, or extracellularly in a soluble form [10]. CD14, upon binding to LPS, facilitates the transfer of LPS to the TLR4/MD-2 complex; studies have demonstrated that this is particularly important in facilitating TLR4 responsiveness to very low levels (<1 ng/ml) of LPS [11]. CD14 has also been implicated in TLR-2 signalling. Studies have demonstrated that CD14 can bind to lipoproteins [12] and potentiate the activation of NF-κB following TLR2/1 ligation [13].

Previous studies in animals have described the presence of TLR2 and TLR4 in the lung and have demonstrated their potential importance in a number of diseases [14]–[16]. However, the expression of TLR2 and 4 and their associated accessory proteins in the peripheral alveolar region of the lung remains unclear. The expression of TLR2, TLR4 and CD14 by alveolar macrophages and monocytes has been previously demonstrated [17], [18]. However, expression of TLR4 and CD14 by the alveolar epithelium is still debated. Studies using the A549 adenocarcinoma cell line as a model of human alveolar type II epithelial cells have demonstrated surface and cytosolic expression of TLR4 and CD14 [19] yet in the absence of serum in the cell culture medium, A549 cells are unresponsive, suggesting an obligate need for exogenous soluble CD14 in order to elicit a TLR4 response [20]. In similar studies we have previously shown that TLR2 and TLR4 mRNA and protein can be detected in primary human alveolar type II epithelial (ATII) cells [21]. However, in marked difference to studies using A549 cells, we have demonstrated that primary human ATII cells are responsive to TLR4 ligation and secrete a number of cytokines and chemokines in the absence of serum [22], [23]. This suggests that, unlike A549 cells, primary human ATII cells express the necessary accessory proteins for TLR4 signalling, although this has not yet been investigated and remains unknown.

To date, studies of the TLR responsiveness of the human alveolar epithelium have focused on ATII cells. Despite being the predominant cell in the alveolar space by number, ATII cells only cover 5% of the surface. The remaining 95% is covered by large attenuated alveolar type I epithelial (ATI) cells. It may therefore be hypothesised that this cell type is more likely to encounter microbes than ATII cells; however, due to their fragile nature it is not possible to isolate these cells from human lung tissue and thus the expression of Toll-like receptors on this cell type has never been investigated. We have recently created and fully characterised an immortalised ATI cell line [24] which allows us to explore the reactivity of this cell type in vitro; thereby giving a unique insight in to the innate immune response of the alveolar epithelium.

In the following study we compared the TLR2 and TLR4 responses of the three major alveolar cell types: macrophages, ATI and ATII cells. In addition we sought to elucidate the role of the accessory proteins CD14 and MD-2 in TLR2 and TLR4 signalling in the alveolus. Our previous studies of LPS-exposed primary human ATII cells and alveolar macrophages show a differential response by these cells to TLR4 activation [22] suggesting that there could be differences in both TLR4 receptor and accessory protein expression and subsequent cell signalling pathways activated in these cells.

We hypothesised that the differential response of alveolar macrophages and ATII cells to TLR2 and TLR4 ligation is due to differential expression of these receptors and their accessory proteins and thus differential activation of MAP kinase signalling pathways. In addition, we suggest that ATI cells are also sensitive to TLR ligands and thus express TLR2 and TLR4 and the necessary accessory proteins.

## Results

### LPS and MALP-2 induce differential mediator release from macrophages, TT1 and ATII cells but not A549 cells

**Figure 1:**
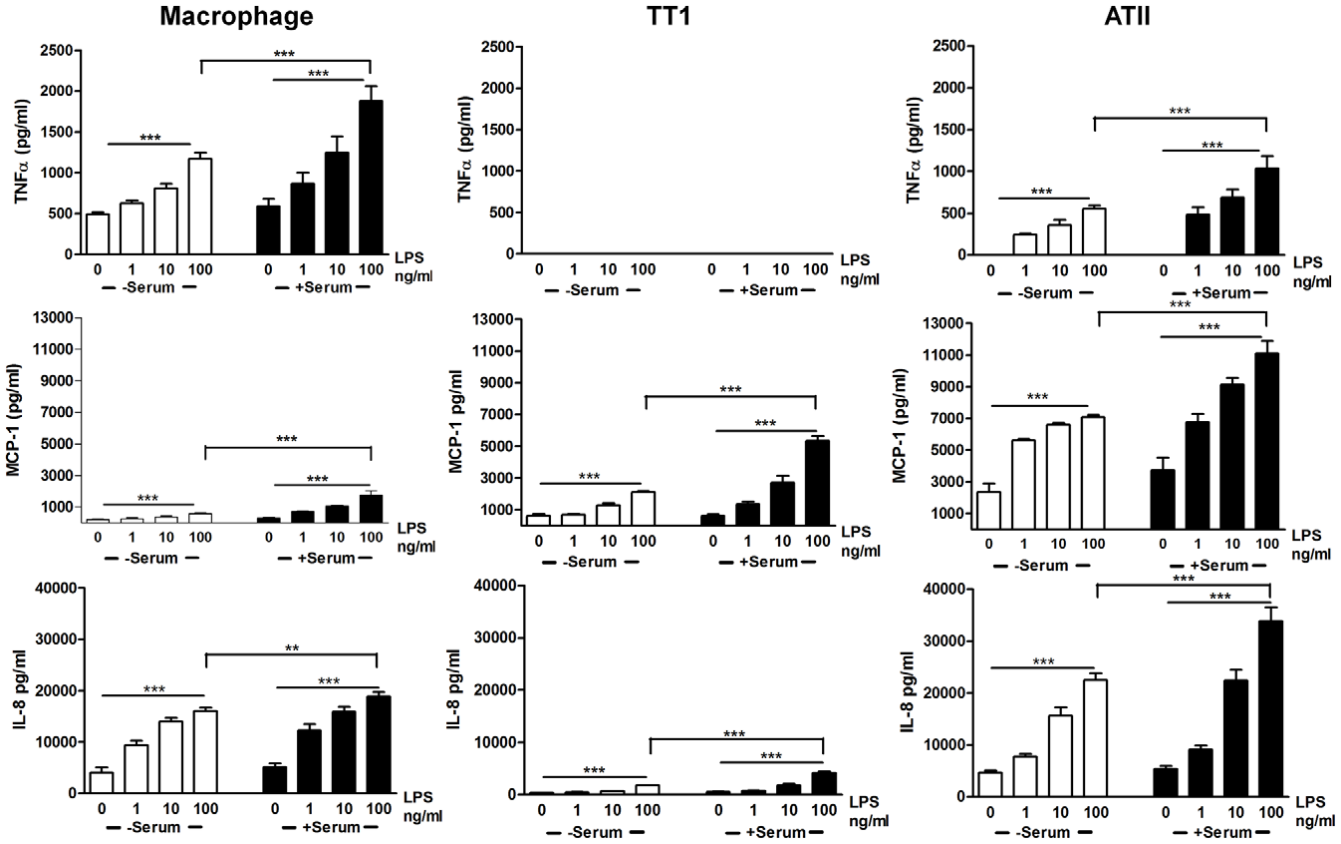
Effect of LPS and serum on cytokine release from alveolar macrophages, ATII and TT1 cells. Cells were exposed to LPS (1–100 ng/ml) in the presence and absence of serum for 24 hours. Conditioned media was then aspirated and TNFα, MCP-1 and IL-8 release measured by Luminex. Data expressed as mean ± SE (n = 6 subjects). **P<0.001, ***P<0.0002.

The effect of increasing concentrations of the TLR4 agonist, LPS, on TNFα, MCP-1 and IL-8 release from primary human alveolar macrophages and TT1 and ATII cells was investigated (Figure 1). LPS caused a concentration dependent increase in release of TNFα (P<0.0001), MCP-1 (P<0.0002) and IL-8 (P<0.0001) from both alveolar macrophages and ATII cells, however only MCP-1 and IL-8 were released from TT1 cells in significant amounts (P<0.0001). TNFα release from alveolar macrophages following LPS exposure was greater than that observed for ATII cells (P<0.02), while MCP-1 release by TT1 and ATII cells was significantly greater than release from alveolar macrophages (P<0.0001). TT1 cells released significantly more MCP-1 than IL-8 in response to LPS (P<0.05) whereas ATII cells released significantly more IL-8 than MCP-1 (P<0.001). As demonstrated by others, A549 cells were not responsive to LPS at the concentrations used in this study either in the absence or presence of serum.

**Figure 2:**
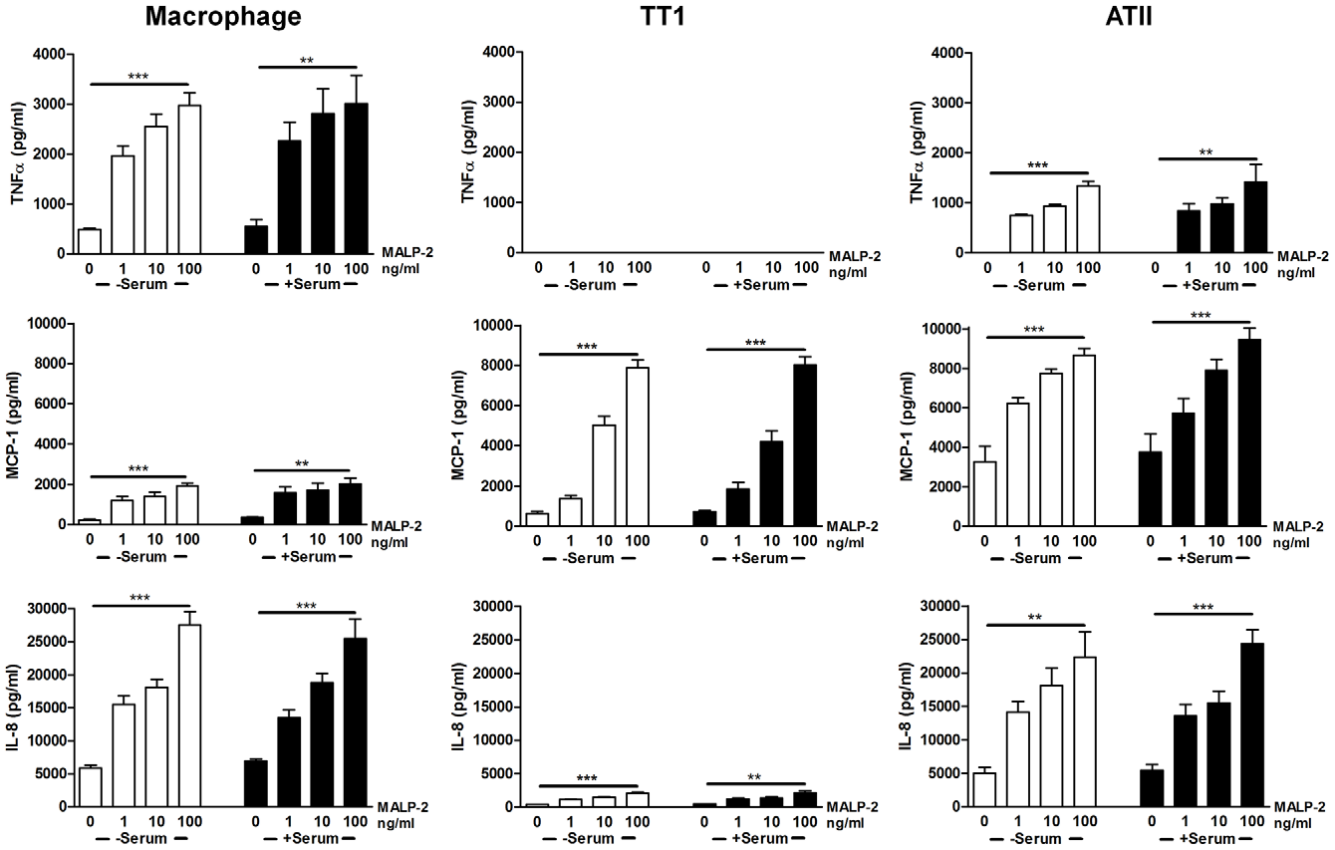
Effect of MALP-2 and serum on cytokine release from alveolar macrophages, ATII and TT1 cells. Cells were exposed to MALP-2 (1–100 ng/ml) in the presence and absence of serum for 24 hours. Conditioned media was then aspirated and TNFα, MCP-1 and IL-8 release measured by Luminex. Data expressed as mean ± SE (n = 6 subjects). **P<0.002, ***P<0.0001.

MALP-2 exposure induced a significant dose dependent increase in MCP-1 (P<0.0001) and IL-8 (P<0.0001) from all three cell types whereas TNFα was only released by alveolar macrophages and ATII cells (P<0.0001; Figure 2). MALP-2 induced a significantly greater release of all three mediators from alveolar macrophages compared to LPS (P<0.002). MALP-2 induced significantly more MCP-1 release from TT1 and ATII cells compared to alveolar macrophages (P<0.0001) whereas release of IL-8 from alveolar macrophages and ATII cells was significantly greater than that from TT1 cells (P<0.0001).

### Serum amplifies the cellular response to LPS but not MALP-2

The addition of serum to the culture medium significantly enhanced IL-8 and MCP-1 release from all three cell types (Figure 1; P<0.001). Similarly, LPS-induced TNFα release from macrophages and ATII cells was also significantly increased in the presence of serum (P<0.0001). As before, LPS did not induce TNFα release from TT1 cells even in the presence of serum. The addition of serum to culture conditions during exposure to MALP-2 did not amplify release of any of the mediators from each of the three cell types (Figure 2).

### Epithelial cells express CD14 but A549 cells do not

**Figure 3:**
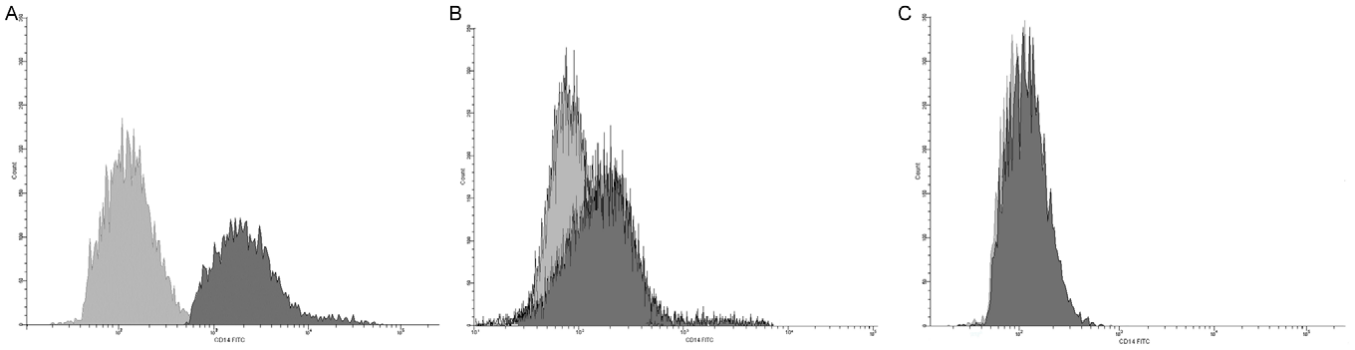
FACS analysis of cell surface CD14 expression on primary human ATII cells, immortalised TT1 cells and A549 cells. Cells were immunostained with FITC-labelled anti CD-14 antibody and expression measured using flow cytometry. Representative hisotgrams demonstrate that ATII cells (A) and TT1 cells (B) express CD14 whereas A549 cells (C) do not.

**Figure 4:**
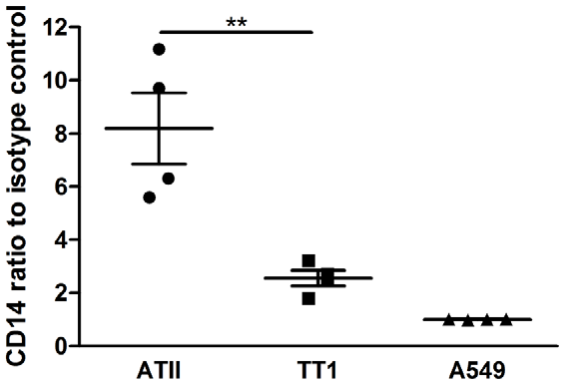
Comparative expression of CD14 on primary human ATII cells, immortalised TT1 cells and A549 cells. Cells were immunostained with CD14 and cells surface expression measured using flow cytometry. The ratio of median fluorescent intensity of CD14 staining relative to IgG control was calculated for all cells. Results demonstrated that both ATII cells and TT1 cells express CD14 whereas A549 cells do not. Furthermore, ATII cells had significantly higher CD14 staining than TT1 cells. Data expressed as mean ± SE (n = 4 subjects). **P<0.007.

Cell surface expression of CD14 was assessed in TT1, ATII and A549 cells using flow cytometry. Both TT1 and ATII cells expressed CD14 whereas A549 cells did not (Figure 3). Furthermore, primary human ATII cells expressed significantly more CD14 than TT1 cells (P<0.007; Figure 4).

### MD2 and CD14 are involved in the LPS but not the MALP-2 response

**Figure 5:**
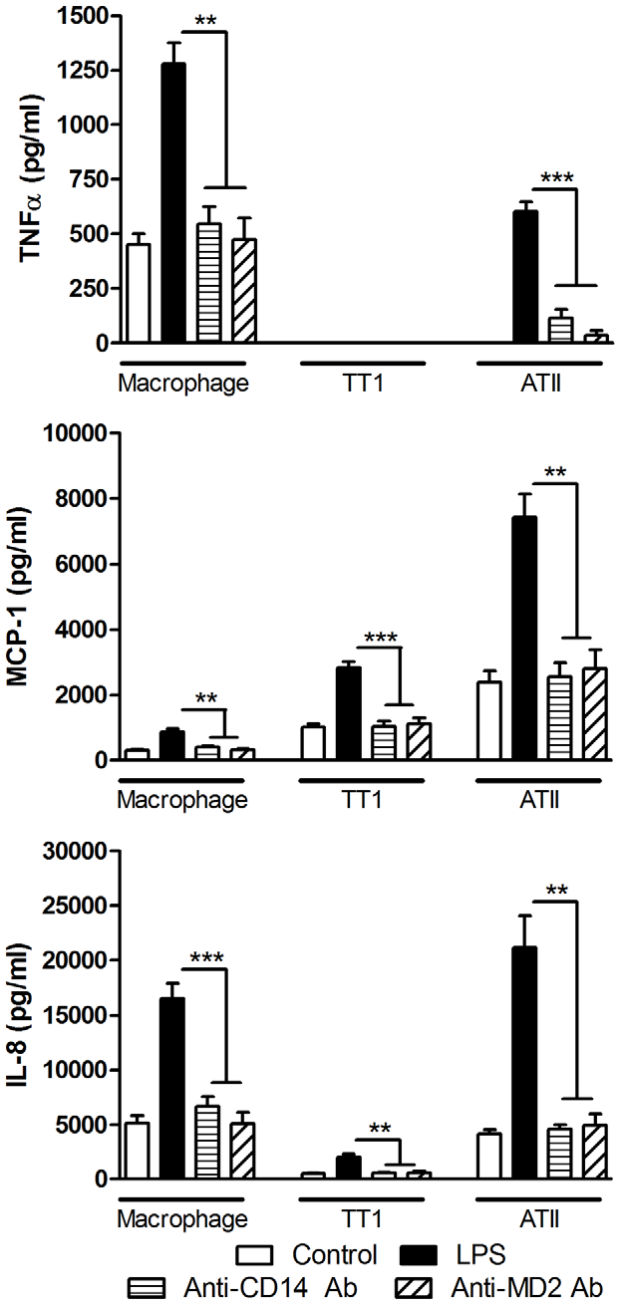
Effect of CD-14 and MD2 neutralisation on LPS-induced cytokine release from alveolar macrophages, ATII and TT1 cells. Cells were pre-incubated with an anti-CD14 or anti-MD2 neutralising antibody for 1 hr before exposure to 100 ng/ml LPS for 24 hours. TNFα, MCP-1 and IL-8 release was measured by Luminex. Data expressed as mean ± SE (n = 4 subjects). **P<0.002, ***P<0.0001. The relevant IgG isotype was used as a control and had no effect on LPS-induced mediator release.

**Figure 6:**
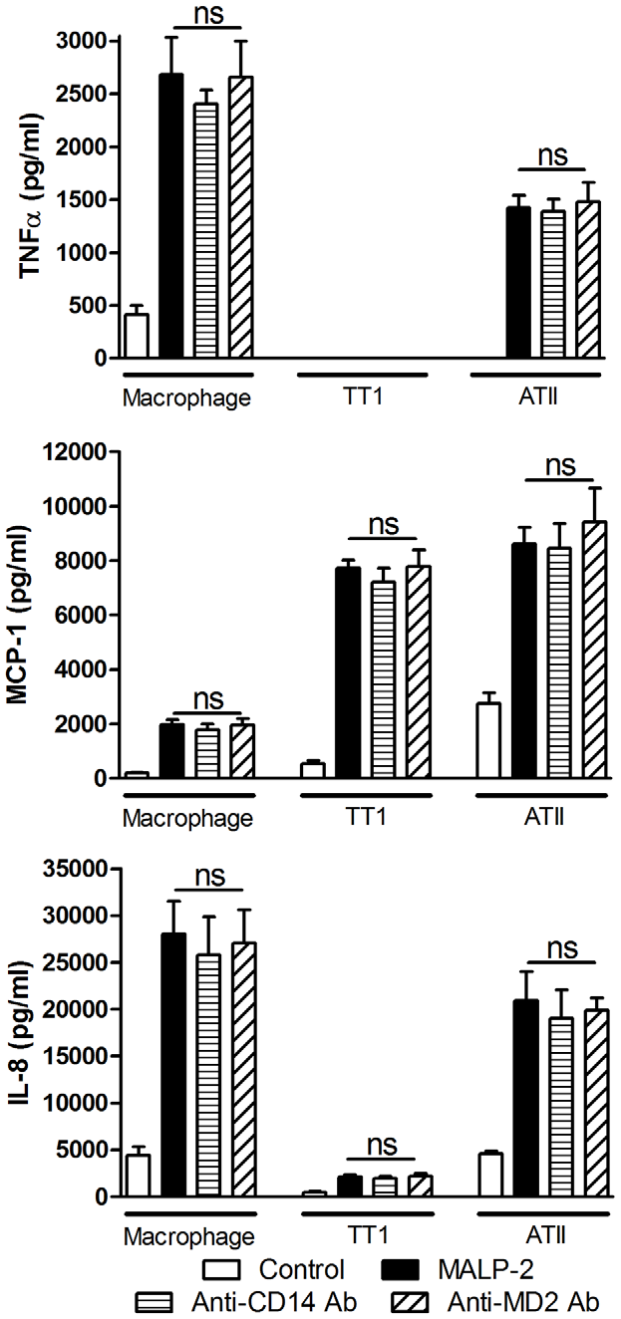
Effect of CD-14 and MD2 neutralisation on MALP-2-induced cytokine release from alveolar macrophages, ATII and TT1 cells. Cells were pre-incubated with an anti-CD14 or anti-MD2 neutralising antibody for 1 hr before exposure to 100 ng/ml MALP-2 for 24 hours. TNFα, MCP-1 and IL-8 release was measured by Luminex. Data expressed as mean ± SE (n = 4 subjects). The relevant IgG isotype was used as a control and had no effect on LPS-induced mediator release.

Neutralisation of MD2 and CD14 significantly inhibited LPS-induced cytokine and chemokine release in all three cell types (P<0.002; Figure 5); almost completely abolishing the LPS response. In contrast, neutralisation of CD14 or MD2 had no effect on MALP-2-induced mediator release from all three cell types (Figure 6). Incubation of cells with the relevant IgG isotype control had no effect on LPS-induced mediator release.

### LPS and MALP-2-induced mediator release involves p38, JNK and ERK

**Figure 7:**
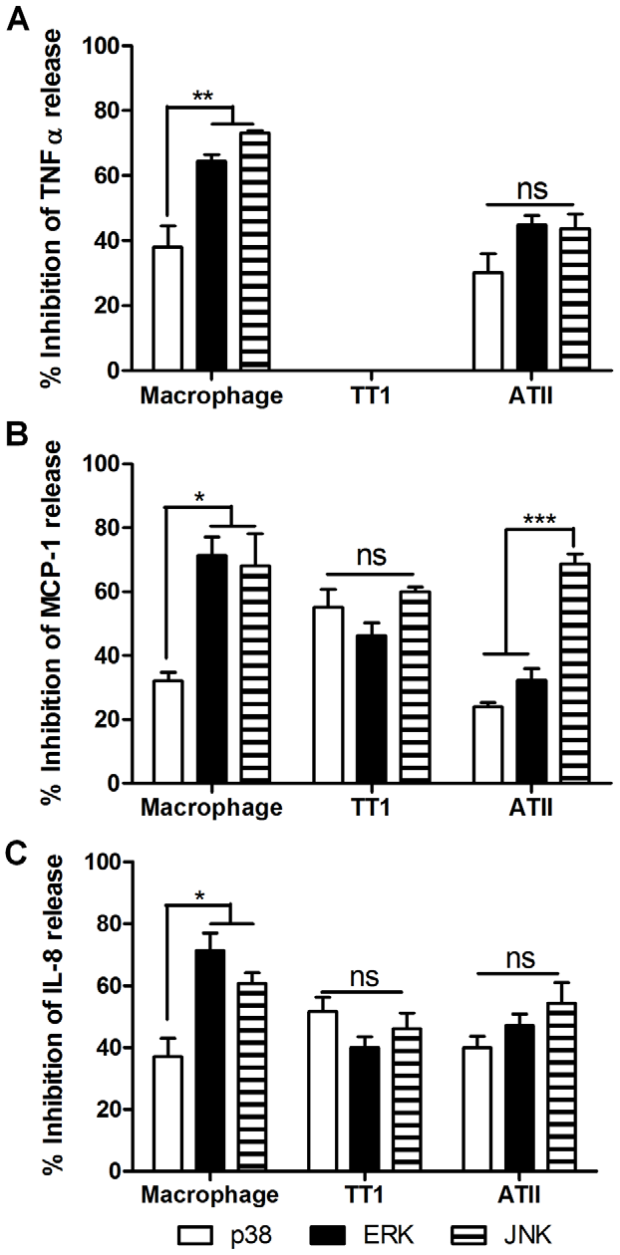
Inhibition of LPS-induced cytokine release from alveolar macrophages ATII and TT1 cells by MAP kinase inhibitors. Cells were pre-incubated for 30 min with 10 µM of inhibitors of p38 (SB202190), JNK (SP600125) or ERK (PD98059) before exposure to 100 ng/ml LPS for 24 hours. TNFα (A), MCP-1 (B) and IL-8 (C) release were measured by Luminex. Data expressed as mean ± SE (n = 4 subjects). *P<0.05, **P<0.002, ***P<0.0002.

**Figure 8:**
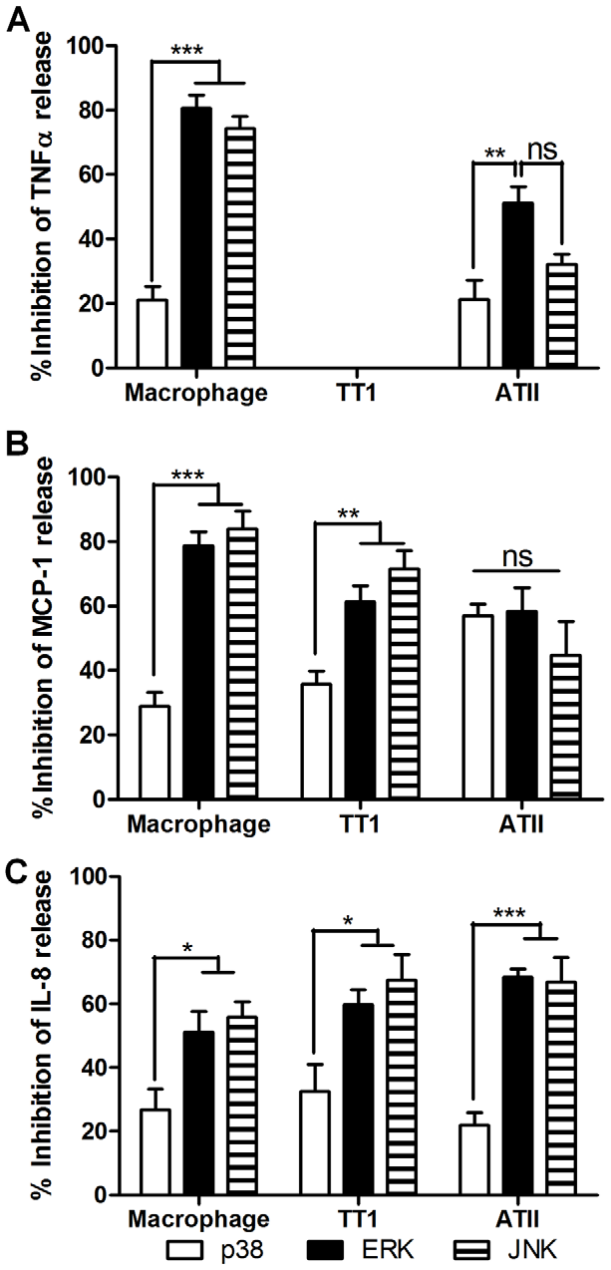
Inhibition of MALP-2-induced cytokine release from alveolar macrophages ATII and TT1 cells by MAP kinase inhibitors. Cells were pre-incubated for 30 min with 10 µM of inhibitors of p38 (SB202190), JNK (SP600125) or ERK (PD98059) before exposure to 100 ng/ml MALP-2 for 24 hours. TNFα (A), MCP-1 (B) and IL-8 (C) release were measured by Luminex. Data expressed as mean ± SE (n = 4 subjects). *P<0.05, **P<0.005, ***P<0.0001.

Inhibition of P38, ERK or JNK all caused an inhibition of LPS (Figure 7) and MALP-2 (Figure 8) -induced cytokine secretion from all three cell types. All three MAP kinase inhibitors had a similar profile of action against LPS-induced TNFα, MCP-1 and IL-8 release from alveolar macrophages; blockade of JNK and ERK caused significantly greater inhibition of mediator release than p38 (P<0.04). Inhibition of ERK also had a significantly greater effect on mediator release from macrophages than TT1 and ATII cells (P<0.05). In ATII cells, the profile of inhibition of the MAP kinases on TNFα, IL-8 and MCP-1 release was markedly different. There was no significant difference in the effect of the inhibitor on TNFα release and IL-8 release from ATII cells or MCP-1 and IL-8 release from TT1 cells. However, Inhibition of JNK had a significantly greater effect on MCP-1 release than p38 and ERK inhibition (P<0.0001) and was significantly more effective at inhibiting MCP-1 than TNFα release (P<0.002).

Similarly to observations following LPS exposure, JNK and ERK inhibitors significantly inhibited MALP-2-induced TNFα, MCP-1 (P<0.0001) and IL-8 (P<0.05) release from alveolar macrophages more than the p38 inhibitor. Similarly JNK and ERK inhibition significantly reduced MALP-2-induced MCP-1 (P<0.005) and IL-8 (P<0.05) more than p38 inhibition. In ATII cells, JNK and ERK inhibitors were significantly more effective than the p38 inhibitor in blocking MALP-2-induced IL-8 release, whereas there was no significant difference between the three inhibitors in inhibiting MCP-1 release.

### LPS and MALP-2 differentially induce TLR4 protein expression by alveolar macrophages, TT1 cells and ATII cells

**Figure 9:**
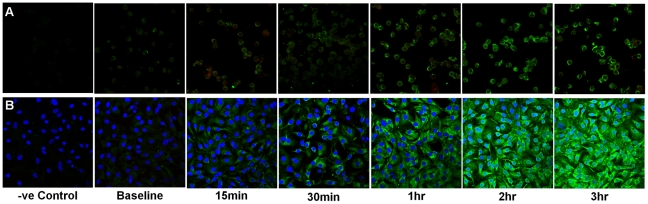
Effect of LPS on alveolar macrophage and ATII cell TLR-4 protein expression. Alveolar macrophages (A) and ATII cells (B) were exposed to LPS (100 ng/ml) over a three hour time course. Cells were fixed in methanol at 0, 15 mins, 30 mins, 1 hr, 2 hrs and 3 hrs and stained with FITC-labelled anti-TLR4 antibody. TLR4 expression was visualised using confocal microscopy. Images were taken through the centre of the cell to visualise cytosolic as well as cell surface expression. Data representative of 3 separate subjects.

**Figure 10:**
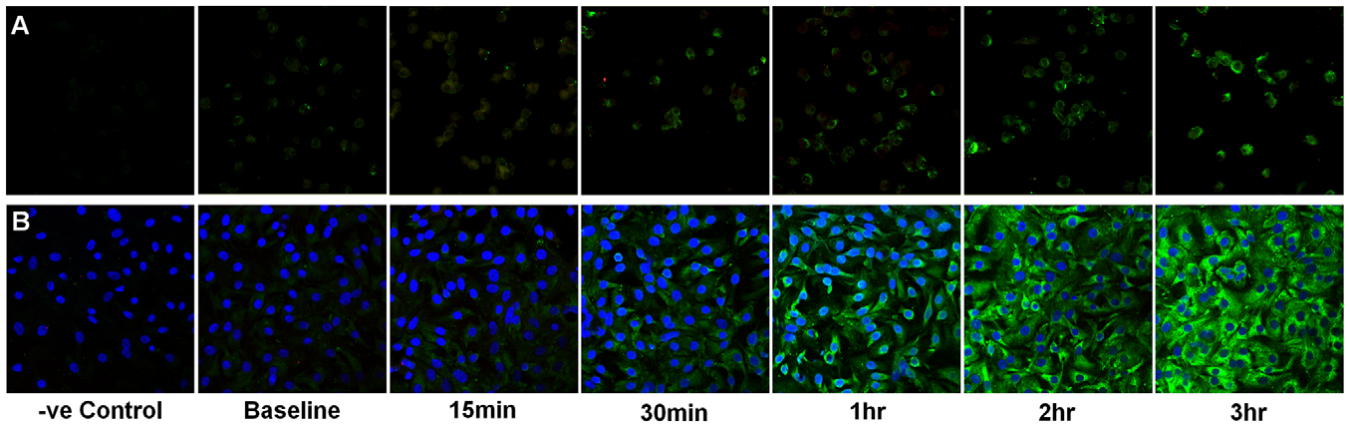
Effect of MALP-2 on alveolar macrophage and ATII cell TLR-4 protein expression. Alveolar macrophages (A) and ATII cells (B) were exposed to MALP-2 (100 ng/ml) over a three hour time course. Cells were fixed in methanol at 0, 15 mins, 30 mins, 1 hr, 2 hrs and 3 hrs and stained with FITC-labelled anti-TLR4 antibody. TLR4 expression was visualised using confocal microscopy. Images were taken through the centre of the cell to visualise cytosolic as well as cell surface expression. Data representative of 3 separate subjects.

**Figure 11:**
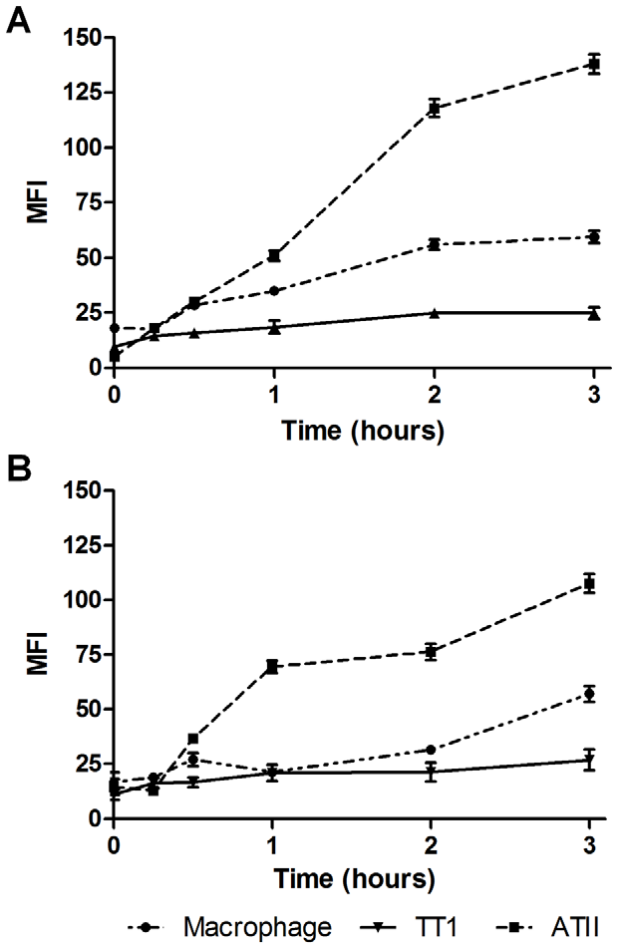
Effect of LPS and MALP-2 exposure on TLR4 expression by alveolar macrophages, ATII and TT1 cells. Using Simple PCI software analysis of images captured by confocal microscopy, the mean fluorescent intensity of staining of individual cells for TLR4 was measured over a 3 hour time course of exposure to LPS (A) or MALP-2 (B). Data expressed as mean ± SE (n = 3 subjects). *P<0.05, **P<0.002, ***P<0.0001.

**Figure 12:**
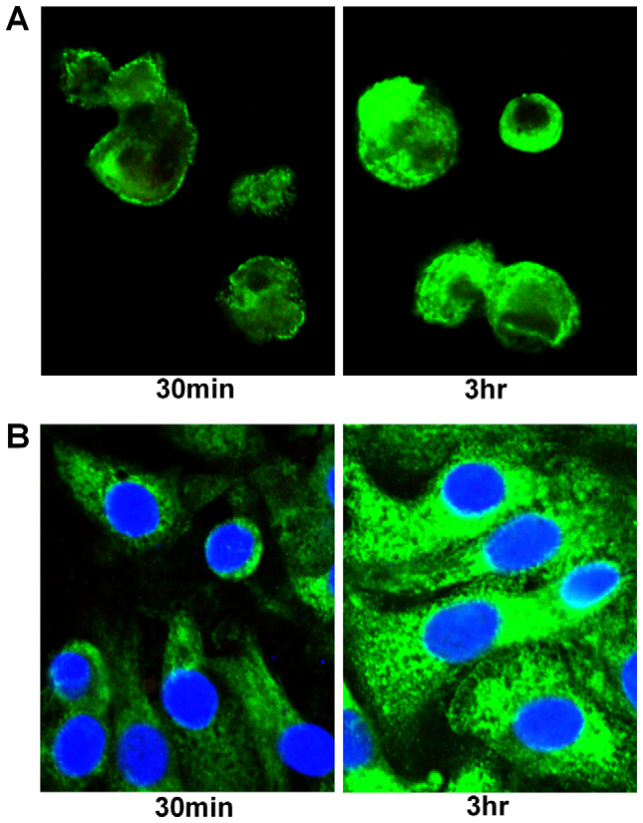
Cellular localisation of TLR4 protein in primary human alveolar macrophages and ATII cells. Under high magnification it can be seen that, in alveolar macrophages (A), there is distinct expression of TLR4 protein at the cell surface at 30 minutes with some intracellular staining. By three hours the intracellular staining has increased greatly (in particular in the perinuclear region). In contrast, in ATII cells there is strong intracellular TLR4 expression at 30 minutes which increases further by 3 hours. Similarly to the alveolar macrophages, intracellular staining is most intense in the perinuclear region. TLR4 expression could not be visualised in ATI cells due to low expression.

LPS and MALP-2-induced TLR expression was measured using image analysis of immunofluorescent confocal images (Figures 9 and 10 respectively). Following exposure to LPS, all three cell types significantly up-regulated TLR4 expression in a time dependent manner (Figure 11A; P<0.0001). ATII TLR4 expression significantly increased after 30 minutes (P<0.05) and by three hours had increased 10-fold (P<0.0001). Immunolocalisation showed that expression of TLR4 in ATII cells was diffuse throughout the cell but appeared most intense in the perinuclear region (Figure 12). When the above experiments were repeated on macrophages, obtained from the same subjects, as well as the TT1 cell line, it was shown that both TT1 and macrophages cells expressed low levels of TLR4 basally. Following LPS exposure, macrophage TLR4 expression increased significantly after 30 minutes (P<0.05) and by three hours was 5.5 fold greater than baseline (P<0.0001). Using confocal microscopy, TLR4 could clearly be seen on the cell surface at early time points as well as intracellularly. However, at later time points expression of the receptors was markedly increased in the cytoplasm and perinuclear region (Figure 12). In TT1 cells expression was significantly greater after 1 hour (P<0.05) and by three hours was 2.5-fold greater than at time zero (P<0.0001).

ATII cells expressed significantly more TLR4 than TT1 cells after one hour exposure (P<0.0001) and alveolar macrophages after two hours (P<0.0001). By two hours, TT1 TLR4 expression was also significantly lower than macrophage expression (P<0.0001). Due to the low level of expression by TT1 cells, it was not possible to clearly visualise the cellular localisation of expression.

Exposure to MALP-2 (Figure 11B) also induced a significant time-dependent increase in TLR4 expression in ATII cells, macrophages (P<0.0001) and TT1 cells (P<0.05). Similar to observations following LPS exposure, TLR4 expression was up-regulated in ATII cells significantly more than macrophages and TT1 cells by one hour (P<0.002). Furthermore, by three hours macrophage TLR4 expression was significantly greater than TT1 cell TLR4 expression (P<0.0002).

Despite using a number of different antibodies and cell staining protocols, it was not possible to visualise TLR2 protein in any of the cells types. It was therefore not possible to quantify the effects of LPS and MALP-2 exposure on TLR2 expression.

## Discussion

These studies demonstrate for the first time, that human alveolar epithelial cells constitutively express CD14 and MD-2, accessory proteins which are essential for TLR4 signalling. We have also demonstrated that LPS and MALP-2, TLR4 and TLR2 ligands respectively, can rapidly induce a marked increase in the expression of TLR4 protein in human alveolar macrophages and ATII cells, and to a lesser extent, TT1 cells and distinct differences in the cytokine responses of these three cell types. This comparison of the innate immune response of primary human alveolar epithelial cells and macrophages obtained from the same subjects provides a unique insight into the differential activation of resident and migratory cells in the respiratory units. Furthermore, the study of a newly generated cell line derived from human alveolar type I epithelial cells, a cell that until now it has not been possible to study in vitro due to its fragile nature preventing it from being isolated in primary culture, further adds to the uniqueness of this study. Thus, we have demonstrated that alveolar epithelial cells can respond to TLR2 and TLR4 ligands in much the same way as do macrophages in an environment relatively low in serum proteins.

Our studies have demonstrated that the profile of cytokine release from alveolar macrophages, TT1 and ATII cells differs in response to TLR2 and TLR4 ligation. We demonstrated that alveolar macrophages were the primary source of TNFα; ATII cells released approximately half the amount of macrophages and TT1 cells released none at all. The observed release of TNFα by ATII cells is somewhat controversial as there are a number of studies suggesting that the alveolar epithelium does not release TNFα [25], [26]. However our results support findings of others demonstrating that under inflammatory and pathological conditions ATII cells from rats [27] and humans [28] can indeed release TNFα. Our study suggests that under basal conditions, macrophages are the sole source of TNFα; however following bacterial colonisation the alveolar epithelium can release TNFα albeit at much lower levels than alveolar macrophages. Following our observations of TLR2 and TLR4-induced TNFα release from primary human ATII cells, we were surprised to discover that TT1 cells did not release any following LPS or MALP-2 exposure. We have previously demonstrated that TNFα can act in an autocrine manner to trigger further release of mediators from ATII cells [22]. It could therefore be postulated that this response may not be desirable in TT1 cells which cover over 95% of the alveolar surface. Such a response in these cells could lead to an excessive inflammatory response that may be deleterious to the host. We also demonstrated that the epithelium is a richer source of chemokines than macrophages following MALP-2 ligation; a finding we have previously demonstrated in ATII cells following LPS exposure [22]. These results further support our hypothesis that leukocyte recruitment during bacterial infection may primarily be due to the robust response of the alveolar epithelium rather than the already resident alveolar macrophages.

Much of the previous research on the innate immune response of the human alveolar region has relied on the use of the A549 adenocarcinoma cell line as a model of ATII cells. These studies have led to the general belief that, in the absence of serum, the alveolar epithelium is unresponsive to LPS despite these cells expressing TLR4 [20]. Our studies using primary human ATII cells and ATI cells immortalised from normal primary human alveolar epithelial cells clearly demonstrate that this is not the case. We demonstrated in our studies that both TT1 and ATII cells are exquisitely sensitive to LPS exposure and release cytokines and chemokines in response to levels as low as 1 ng/ml even in the absence of serum. One of the primary beliefs for the lack of LPS responsiveness and the obligate need for serum (a source of soluble CD14) is that cells of an epithelial origin do not constitutively express functional levels of CD14 [29], [30]. We have confirmed this in our studies; using flow cytometry we demonstrated that the A549 adenocarcinoma cells line does not express CD14. However, a significant finding in our studies is that both TT1 cells and primary human ATII cells express CD14 constitutively, and that neutralisation of CD14 with antibodies blocks LPS-induced release of IL-8 and MCP-1. This was also the case for alveolar macrophages. Despite epithelial expression of CD14 we do however demonstrate that the addition of serum, a rich source of soluble CD14, further amplifies the response to LPS. This ability of exogenous soluble CD14 may be particularly important in conditions such as acute lung injury where there is significant microvascular leakage in to the alveolar space.

In these studies we also investigated the effect of serum and CD14 neutralisation on MALP-2 induced mediator release as there is currently debate as to whether CD14 is involved in facilitating TLR-2 signalling. It is widely accepted that CD14 is involved in the signalling of triacylated lipoproteins through the TLR2/1 heterodimer [13] however studies of the role of CD14 in signalling of diacylated lipoproteins through TLR2/6 have yielded opposing views [12], [31]. Our studies demonstrated that the addition of serum to the culture medium did not potentiate MALP-2-induced mediator release and CD14 neutralisation did not inhibit MALP-2-induced mediator release from any of the three cell types. These results confirm findings that diacylated lipoproteins do not require CD14 to signal through the TLR2/6 complex.

In addition to the role of CD14 in TLR signalling we also investigated the role of MD-2 in the responsiveness of macrophages and the alveolar epithelium to LPS and MALP-2 stimulation. Our results demonstrated that neutralisation of MD-2 completely abolished the LPS response in all three cell types. This confirms previous studies demonstrating the importance of MD-2 in TLR4 signalling. Crystal structure analysis of the LPS/MD-2/TLR4 complex has shown that binding of LPS to MD-2 is essential for receptor dimerisation and therefore subsequent signalling [32]. Furthermore, functional in vitro studies of intestinal epithelial cells have also demonstrated that MD-2 is solely responsible for LPS responsiveness [33]. Unsurprisingly, we also demonstrated that MD2 was not involved in TLR2 signalling but these studies helped demonstrate the specificity of MD2 neutralisation on TLR4 signalling.

We have previously demonstrated that LPS activates MAP kinases in ATII cells and macrophages [22]. However, in order to elucidate the relative role of each of the MAP kinases in mediator production following TLR2 and TLR4 activation, alveolar macrophages, TT1 and ATII cells were pre-treated with inhibitors of p38, JNK and ERK before being exposed to LPS and MALP-2. In all experiments, inhibition of JNK or ERK was more effective at inhibiting cytokine secretion from macrophages compared to p38. Nevertheless, p38 was found to contribute, although the data suggest that it plays a relatively smaller role. This in contrast to other very recent studies [34] suggesting that ERK and p38 play a similar role in LPS-induced TNFα secretion by human alveolar macrophages. However, this difference in results may be due to the presence of serum in the aforementioned study. Studies investigating the effect of serum on MAP kinase activation in the THP-1 monocyte cell line show that the presence of serum increases MAP kinase phosphorylation, in particular p38 [35].

In our studies of the role of MAP kinase activation in mediator release from TT1 and ATII cells, we demonstrated that all three MAP kinases played a significant role in mediator release. JNK was particularly important in TLR4-mediated MCP-1 release from ATII cells, whereas JNK, ERK and p38 contributed to a similar degree in TLR4-mediated TNFα and IL-8 release. Similarly, in TT1 cells, all three MAP kinases contributed to LPS-induced MCP-1 and IL-8 release to a similar degree. Following TLR2 ligation, JNK appeared to play a significantly greater role than p38 in MCP-1 and IL-8 release from TT1 cells and IL-8 from ATII cells. Similarly, ERK played a significantly greater role in TNF and IL-8 release from ATII cells than p38. These results suggest that JNK and ERK may be more effective targets than p38 for anti-inflammatory therapy during bacterial infection.

It is interesting to note that, inhibition of ERK generally had a greater effect on macrophages than TT1 and ATII cells. We hypothesise that the differences in the profile of inhibition of mediator release from ATII cells compared to macrophages may be due to alternative pathways being activated following TLR ligation. Previous studies in our laboratory have shown that a significant proportion of IL-6 released following LPS stimulation of ATII cells is dependent on feedback control by LPS-induced IL-1β and TNFα release, which was not the case in macrophages [22]. Subsequent ligation of the IL-1 and TNF receptors may lead to activation of pathways that induce TNFα and MCP-1 release via NF-κB. Studies using human epithelial cell lines [36] and kidney cells [37] have demonstrated that IL-1β and TNFα activate NF-κB via pathways that LPS is unable to.

In addition to the TLR2 and TLR4-induced signalling events and cytokine and chemokine release, we have also investigated the effect of TLR ligation on receptor protein expression and cellular localisation, which until now, has been unclear. In the present study, we demonstrated up-regulation of TLR4 receptors at the protein level within three hours of exposure to LPS and MALP-2 in all three cell types, in particular alveolar macrophages and ATII cells. The relative lack of increased expression of TLR4 in TT1 cells following LPS exposure may be a protective mechanism in order to prevent an exaggerated inflammatory response. As already mentioned, TT1 cells cover 95% of the alveolar surface and are therefore most likely to encounter bacteria. The relatively restrained immune response of TT1 cells to LPS exposure may be sufficient to trigger an inflammatory response in the surrounding ATII cells and macrophages but not sufficient enough to cause deleterious effects from too robust an inflammatory response. Over the time course of exposure, the signal for TLR4 protein expression, which was detected in the outer cell membrane and intracellularly in macrophages, and primarily intracellularly in ATII cells, was found to increase rapidly over time, particularly intracellularly in the perinuclear region, a process that has also been demonstrated in monocytes [38]. It may be hypothesised that intracellular expression of TLR4 may serve to regulate cellular responsiveness to LPS.

It is well understood that following binding of LPS to TLR4 at the cell surface, MyD88-dependent signalling pathways are activated that result in MAP kinase activation and cytokine secretion [39]. In addition, recent studies have demonstrated that TLR4, once bound to LPS and internalised, can go on to activate the IRF3 pathway [40]. The ability of TLR4 to signal intracellularly may explain why cells that express high levels of TLR4 intracellularly, such as human ATII cells discussed herein, can respond to LPS even though there is apparently little surface expression. Studies of the BEAS2B epithelial cell line also demonstrated that TLR4 was expressed exclusively intracellularly and that exposure to LPS activated MAP kinases and induced cytokine secretion [41]. However, in these studies, levels exceeding 1 µg/ml LPS were required to elicit a robust cytokine response and LPS exposure did not induce further TLR4 expression. This is in marked difference to our results and suggests that there may be differences between the two cell types. From our TLR4 and CD14 expression studies we hypothesise that ATI and ATII cells are able to respond to LPS both at the cell surface and intracellularly although this needs further investigation to confirm the exact cellular location at which signalling occurs.

A particularly interesting observation in this study was that TLR2 ligation resulted in up-regulation of TLR4 protein. The interplay between TLR2 and TLR4 expression during infection has been demonstrated in in vivo and in vitro models [42], [43]. However, these studies have primarily focused on the effect of TLR4 ligation on TLR2 expression. We believe this to be the first study to show TLR2 ligation up-regulates TLR4 expression in human alveolar macrophages and epithelial cells. Such a mechanism may play an important role in sepsis and in chronic lung diseases where repeated infection is common, such as COPD and cystic fibrosis.

We were disappointed to not be able to detect TLR2-protein despite using a number of different antibodies and staining protocols. All three cell types respond to MALP-2, a TLR-2/6 specific ligand so must express the receptors. This suggests that the receptors are expressed at low levels that cannot be easily detected but are still capable of eliciting a robust response or that the antibodies available are not suitable for this technique.

In conclusion, we have shown for the first time that the human alveolar epithelium expresses functional CD14, MD-2 and TLR4 and responds to TLR2 ligands to produce a robust innate immune response. Furthermore these studies of primary human alveolar macrophages and ATII cells from the same subjects and immortalised ATI cells demonstrate the dynamic nature of the TLR signalling system. Thus, a single ligand can elicit distinct responses in different cell types, following activation of one type of TLR, via different cell signalling pathways and up-regulation of identical TLRs. Together with our previous observations, that the cytokine feedback control mechanisms are also different between ATII and macrophages, we propose that the rapid response of the respiratory unit to microbial infection critically depends on co-ordinated action of these cells to initiate the innate immune response.

## Methods

### Ethics statement

The tissue used in this study was surplus tissue obtained following resection for lung carcinoma. Written informed consent was obtained for all samples and the study was carried out with the approval of the Royal Brompton and Harefield Ethical Committee (Ref: 08/H0708/73).

### Isolation of primary human alveolar macrophages and type II epithelial cells

ATII cells and macrophages were isolated from lung tissue as previously described (n = 21 samples; [23]). Briefly, lung sections were perfused by injection of sterile saline until the cell count was less than 1×10^4^ cells/ml. The draining lavage was then collected and centrifuged (290 g, 10 min, 20°C). The cell pellet was re-suspended in serum free DCCM-1 (Cadama, UK) containing 1% penicillin/streptomycin/glutamine (PSG; Invitrogen, Paisley, UK) and plated in 24 well tissue culture plates (VWR, Lutterworth, UK) at a density of 0.5×10^6^ macrophages/well. After three hours macrophages had adhered and the medium was removed and the wells were washed to remove non-adherent cells. The macrophages were maintained in serum free DCCM-1+1% PSG. We have shown previously that >95% of these cells are CD68+, phagocytic and show macrophage morphology on scanning electron microscopy [23], [44].

To isolate ATII cells, tissue was perfused and inflated with trypsin (0.25% in HBSS; Sigma, Poole, UK) and incubated at 37°C for 45 minutes; trypsin was replaced twice during this time. The tissue was finely chopped in the presence of newborn calf serum (NCS; Invitrogen). The chopped tissue was then incubated with DNase (250 µg/ml; Sigma) and the mixture passed through a 300 µm filter, followed by a 40 µm filter to remove large tissue debris. The cell suspension was then centrifuged (290 g, 10 min, 20°C) and the resulting pellet re-suspended in DCCM-1 medium containing 50 µg/ml DNase. These cells were incubated in tissue culture flasks for two hours at 37°C in a humidified incubator to allow differential adherence of contaminating mononuclear cells.

After two hours the non-adherent ATII cells were removed and the cell suspension centrifuged as before. The cell pellet was then re-suspended in DCCM-1 containing 10% NCS and 1% PSG at a concentration of 0.5×10^6^ cells/ml. Cells were then seeded at 0.5×10^6^ ATII cells per well in 24 well plates coated in 1% PureCol solution (PureCol, Leimuiden, Netherlands; Type I collagen). By 48 hours, cells had become confluent and had a purity of >95% as previously demonstrated [45]. These cells have been thoroughly characterised using electron microscopy, which shows that the cells are cuboidal in morphology, have surfactant-containing lamellar bodies, tight junctions and microvilli. Furthermore they stain positively for the ATII cell marker alkaline phosphatase and express surfactant proteins A and C and maintain their phenotype for up to six days [45],[46].

### Culture of immortalised ATI cells

We have created an immortalised human ATI cell line [24], using previously published techniques [47], that shows the same characteristics as ATI cells in vivo. Stocks of TT1 (immortalised ATI) cells were plated in DCCM-1 media containing 10% NCS at a density of 0.5×10^6^/well in a 24 well plate. Cells reached confluency within 48 hrs.

### Culture of A549 cells

Frozen stocks of A549 cells were thawed and re-suspended in DCCM-1 containing 10% NCS at a density of 0.5×10^6^/well in a 24 well plate. Cells reached confluency within 48 hrs. Upon reaching confluency cells were serum starved for 24 hours before experiments were carried out.

### Expression of CD14

Epithelial CD14 expression was measured by FACS using as previously described [48]. 1×10^6^ cells were incubated for 1 hr with FITC-conjugated anti-CD14 or IgG2_a_ isotype control antibody (50 µg/ml; BD Biosciences, UK). Cells were then washed and re-suspended in FACSFlow™ (BD Biosciences) prior to analysis. Ten thousand events were acquired and the data presented as the relative fluorescence, which was calculated by the fluorescence values (median channel) of the cells stained with FITC-conjugated anti-CD14 divided by the fluorescence values (median channel) for the IgG2a isotype control.

### Stimulation of TLR2 and TLR4 in the presence and absence of serum

Following 24 hrs serum starvation, TT1, ATII cells and macrophages (n = 6 subjects/cell passages) were exposed to ultrapure LPS (1, 10, 100 ng/ml; Escherichia coli 055:B5; Axxora, UK) or MALP-2 (1, 10,100 ng/ml; Axxora, UK) in serum-free media or media containing 10% endotoxin free NCS (Invitrogen, UK) for 24 hours.

### Neutralisation of CD14 and MD2

Cells were incubated for 1 hr with 10 µg/ml neutralising polyclonal rabbit anti-human MD-2 (AbD serotec, UK) and monoclonal anti-human CD14 (clone 61D3; AbCam, UK) antibodies prior to addition of 100 ng/ml LPS or MALP-2 for 24 hrs as described above (n = 4). The corresponding IgG isotypes were used as negative controls (Abcam, UK).

### Inhibition of MAP kinases

We have previously demonstrated LPS-induced activation of MAP kinases in ATII cells and macrophages [22]. To assess the contribution of MAP kinases to TLR2 and TLR4-mediated cytokine secretion, cells were incubated for 30 minutes with 10 µM of commercially available inhibitors to; p38 (SB202190), ERK (PD98059) and JNK (SP600125; Cambridge Bioscience, UK) prior to the addition of 100 ng/ml LPS or MALP-2 for 24 hours.

### Measurement of TNFα, MCP-1 and IL-8 release

Cytokine secretion was measured using the Luminex Beadlyte™ human multi-cytokine detection system (Millipore, UK). Assay samples were run in triplicate as per the manufacturer's protocol. Sample concentrations were calculated from a standard curve using a five-parameter regression formula.

### Immunofluorescent labelling of TLR2 and TLR4

Alveolar macrophages, ATII cells and TT1 cells (n = 3) were cultured on coverslips and exposed to LPS or MALP-2 (100 ng/ml) over a time course of 3 hours. At each time point (0, 15 min, 30 min, 1 hr, 2 hrs and 3 hrs) cells were fixed and permeabilized in cold methanol. Following this, coverslips were blocked using PBS containing 1% BSA (Sigma) for 1 hour at room temperature. Cells were then incubated with either PE-labelled mouse anti-TLR-2 or FITC-labelled mouse anti-TLR-4 (AMS Biotechnology, Abingdon, UK) at a concentration of 5 µg/ml for two hours at room temperature. Cells were then washed three times in PBS for 5 minutes and prepared for nuclear staining. As a negative control, cells were incubated with the appropriate IgG carrier protein (Dako) as described above to rule out any non-specific effects of the staining procedure.

To stain the nucleus, 300 µl of DAPI stain (Sigma) at a concentration of 1 µg/ml was added to each well for one minute. The DAPI was then aspirated and the cells were washed as before. Coverslips were mounted on microscope slides in 4.5 µl of Citifluor (Agar Scientific, Stansted, UK) and covered with a glass coverslip. In order to visualise intracellular staining of TLRs, confocal images were taken through the centre of the cells following a scan of the whole cell layer through the Z plane.

### Quantification of TLR expression

TLR expression in macrophages, TT1 and ATII cells was measured using SimplePCI software (Digital Pixel, UK). Using images captured by fluorescent microscopy the increase in TLR2 and TLR4 expression was assessed by measuring the mean fluorescent intensity (MFI).

### Statistical analyses

Data are presented as mean ± SE. A two-way ANOVA with Bonferroni multiple comparison post-test was used to analyse the concentration-dependent effects of MALP-2 and LPS in the presence and absence of serum on cytokine and chemokine release from TT1, ATII cells and alveolar macrophages. A two-way ANOVA with Bonferroni multiple comparison post-test was also use to compare the effect of TLR ligation on TLR4 expression over time in the three cell types. A one-way ANOVA with Bonferroni multiple comparison post-test was used to compare the effect of MAP kinase inhibitors in each of the cell types. Unpaired *t* tests were used to determine significant differences in CD14 and MD2 neutralisation in each cells type and compare CD14 expression in the three cell types. A *P* value of <0.05 was considered to be statistically significant.
